# CRISPR Interference-Mediated Silencing of the *mmpL3* Gene in *Mycobacterium smegmatis* and Its Impact on Antimicrobial Susceptibility

**DOI:** 10.3390/antibiotics13060483

**Published:** 2024-05-24

**Authors:** Yonita Yuliani, Azizah Fitriana Nurul Ilmi, Suthidee Petsong, Ajcharaporn Sawatpanich, Sunisa Chirakul, Tanittha Chatsuwan, Tanapat Palaga, Suwatchareeporn Rotcheewaphan

**Affiliations:** 1 Medical Sciences, Faculty of Medicine, Chulalongkorn University, Bangkok 10330, Thailand; yonitayuliani21@gmail.com (Y.Y.); ilmi.azizah@hotmail.com (A.F.N.I.); 2 Department of Microbiology, Faculty of Medicine, Chulalongkorn University, Bangkok 10330, Thailand; spsuthidee@gmail.com (S.P.); ajcharaporn.pat@gmail.com (A.S.); chirakulsunisa@gmail.com (S.C.); smedtcs@hotmail.com (T.C.); 3 Center of Excellence in Antimicrobial Stewardship, Chulalongkorn University, Bangkok 10330, Thailand; 4 Department of Microbiology, Faculty of Science, Chulalongkorn University, Bangkok 10330, Thailand; tanapat.p@chula.ac.th

**Keywords:** *Mycobacterium smegmatis*, antimicrobial susceptibility, CRISPRi, *mmpL3*, essential gene

## Abstract

Background: The discovery of novel therapeutic agents, especially those targeting mycobacterial membrane protein large 3 (*mmpL3*), has shown promise. In this study, the CRISPR interference-*Streptococcus thermophilus* nuclease-deactivated Cas9 (CRISPRi-dCas9_Sth1_) system was utilized to suppress *mmpL3* expression in *Mycobacterium smegmatis*, and its impacts on susceptibility to antimicrobial agents were evaluated. Methods: The repression of the *mmpL3* gene was confirmed by RT-qPCR. The essentiality, growth curve, viability, and antimicrobial susceptibility of the *mmpL3* knockdown strain were investigated. Results: *mmpL3* silencing was achieved by utilizing 0.5 and 1 ng/mL anhydrotetracycline (ATc), resulting in reductions in the expression of 60.4% and 74.4%, respectively. *mmpL3* silencing led to a significant decrease in bacterial viability when combined with one-half of the minimal inhibitory concentrations (MICs) of rifampicin, rifabutin, ceftriaxone, or isoniazid, along with 0.1 or 0.5 ng/mL ATc (*p* < 0.05). However, no significant difference was observed for clarithromycin or amikacin. Conclusions: The downregulation of the *mmpL3* gene in mycobacteria was achieved through the use of CRISPRi-dCas9_Sth1_, resulting in growth deficiencies and resensitization to certain antimicrobial agents. The impact was dependent upon the level of gene expression.

## 1. Introduction

Mycobacterial infections have emerged as a significant global health problem, primarily due to the increase in the prevalence of drug-resistant *Mycobacterium tuberculosis* and drug-resistant nontuberculous mycobacteria (NTM) in various regions. These drug-resistant strains present a substantial challenge, as they diminish the efficacy of commonly used antimicrobial agents in treatment regimens. To address this challenge, the discovery of new therapeutic agents and targets for effective pathogen elimination is important. An example of an essential gene of interest is mycobacterial membrane protein large 3 (*mmpL3*), responsible for transporting trehalose monomycolate, which serves as a precursor for producing trehalose dimycolate and mycolate-bound arabinogalactan within the cell [1,2]. The efficacy of *mmpL3* inhibitors against *M. tuberculosis*, such as SQ109 [3,4], NITD-304, NITD-349, AU1235, and AU36 [5], and NTM, such as PIPD1 [6], has been established, demonstrating their effectiveness as antimycobacterial agents.

Combination drug regimens are recommended for mycobacterial infections, including the use of rifampicin, isoniazid, ethambutol and pyrazinamide for tuberculosis [7], as well as macrolides, aminoglycosides, ethambutol, and rifampicin for NTM infections [8]. Nevertheless, mycobacteria exhibit intrinsic resistance to diverse classes of antimicrobial drugs, such as rifampicin in *M. smegmatis* and β-lactams in both *M. smegmatis* and *M. tuberculosis* [9,10,11,12]. Furthermore, acquired resistance to these antimicrobial drugs further limits treatment options. Therefore, enhancing the efficacy of existing antimicrobial drugs that are currently ineffective is imperative for managing and eradicating mycobacterial infections. Previously, the synergistic effects of MmpL3 inhibitors [4,5,13,14] or *mmpL3* knockdown [15,16,17] and certain antimicrobial agents have been investigated. However, these studies demonstrated different susceptibility results depending on the experimental conditions.

One promising tool for studying mycobacterial genes is the clustered regularly interspaced short palindromic repeats interference (CRISPRi) system. This system has been developed to facilitate the manipulation and regulation of gene silencing in *Mycobacterium*. Specifically, the CRISPRi system utilizes *Streptococcus thermophilus* nuclease-deactivated Cas9 (dCas9_Sth1_) to effectively suppress the expression of endogenous mycobacterial genes [18]. The CRISPRi-dCas9_Sth1_ system demonstrates both specificity and efficiency in the gene knockdown of essential and nonessential mycobacterial genes, including those associated with drug resistance [19,20]. In this study, we investigated the impact of silencing the mycobacterial *mmpL3* gene via the CRISPRi-dCas9_Sth1_ system on susceptibility to different classes of non-MmpL3-dependent antimicrobial agents, including rifampicin, rifabutin, isoniazid, amikacin, clarithromycin, and β-lactam drug. A comprehensive understanding of how a reduction in *mmpL3* expression influences antimycobacterial susceptibility is crucial for the advancement of therapeutic strategies.

## 2. Results

### 2.1. CRISPRi Targeting of the mmpL3 Gene Impacts M. smegmatis Growth

**Figure 1:**
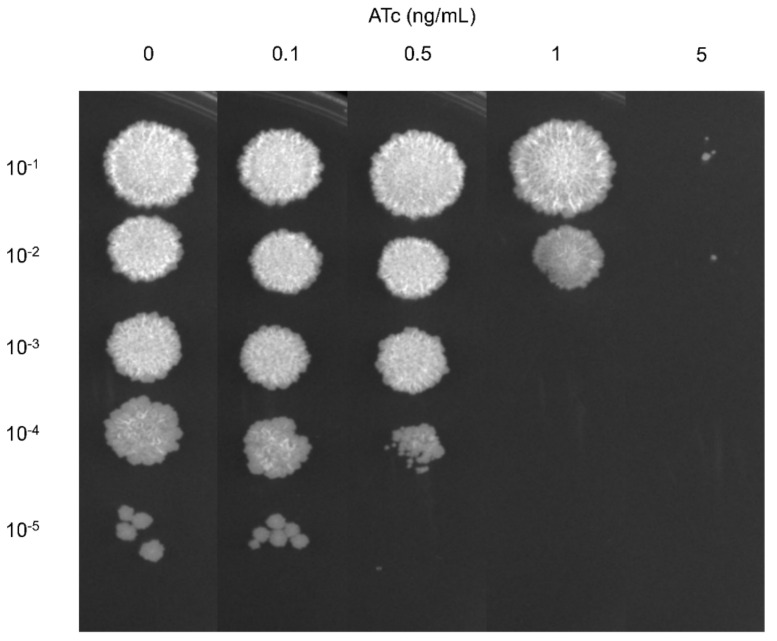
Essentiality of the *mmpL3* gene in the *mmpL3*_KD *M. smegmatis* strain. Serial dilutions (10^−1^ to 10^−5^) of log-phase cultures of the *mmpL3*_KD *M. smegmatis* strain were plated on 7H10 agar in both the absence and presence of varying concentrations of ATc (0.1, 0.5, 1, or 5 ng/mL).

The *mmpL3* knockdown *M. smegmatis* (*mmpL3*_KD) strain was constructed to verify the essentiality of the *mmpL3* gene using the CRISPRi-dCas9_Sth1_ system which interferes the transcription elongation from binding of dCas9– single guide RNA (sgRNA) complex to *mmpL3* gene [18]. The specificity of sgRNA targeting *mmpL3* gene [21] was verified using BLAST tool (NCBI) against the genome of *M. smegmatis* MC^2^155. No complementarity of the *mmpL3* sgRNA sequence was identified in the other sites of the genome of *M. smegmatis*. The sgRNAs targeting the *mmpL3* gene effectively suppressed the growth of the *mmpL3*_KD strain in a dose-dependent manner at anhydrotetracycline (ATc) concentrations ranging from 0.5 to 50 ng/mL, while the pLJR962 control strain exhibited normal growth (Figure 1 and Supplementary Figure S1). Mycobacterial growth of *mmpL3*_KD was completely inhibited at ATc concentrations of 5, 10, and 50 ng/mL. There was no apparent impairment of mycobacterial growth in the presence of 0.1 ng/mL ATc. Furthermore, a few ATc-resistant strains were observed on 7H10 agar plates containing high concentrations of ATc (5, 10, or 50 ng/mL). These ATc-resistant colonies were capable of growing upon subculture on agar containing ATc. Moreover, induction with ATc did not result in toxicity, as evidenced by the normal growth of the pLJR962 control strain (Supplementary Figure S1).

### 2.2. mmpL3 Gene Expression in the Presence and Absence of ATc

**Figure 2:**
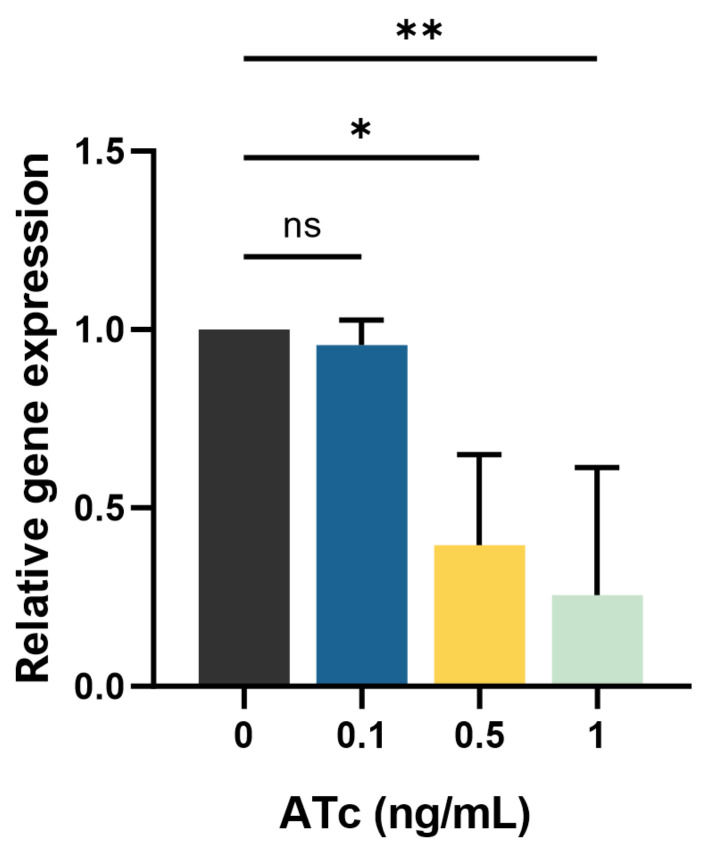
*mmpL3* gene expression in the *mmpL3*_KD *M. smegmatis* strain in the presence and absence of ATc (ng/mL). The experiments were conducted in biological and technical triplicates. Statistical significance is indicated as * *p* < 0.05 and ** *p* < 0.01, and “ns” indicates not statistically significant. The error bars represent the SDs of the means.

The impact of inducing the transcription response on *mmpL3*_KD expression in the *M. smegmatis* strain was assessed using RT-qPCR. *mmpL3* expression in *mmpL3*_KD decreased upon induction with 0.1, 0.5, and 1 ng/mL ATc, resulting in reductions in the expression of 4.4% ± 7.1% (mean ± standard deviation (SD)), 60.4% ± 25.5%, and 74.4% ± 35.7%, respectively. Compared to that in the absence of ATc, *mmpL3* expression was significantly lower in the presence of 0.5 and 1 ng/mL ATc, with *p* < 0.05 and *p* < 0.01, respectively (Figure 2).

### 2.3. Growth Curve and Viability of mmpL3_KD M. smegmatis at Different ATc Concentrations

**Figure 3:**
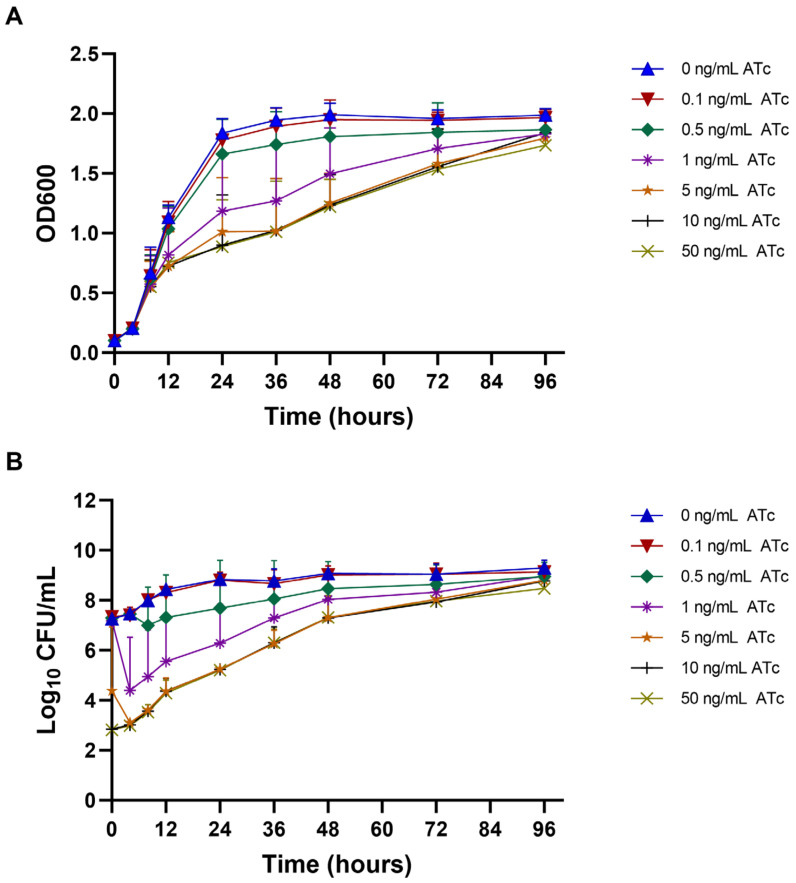
Growth curve and viability of the *mmpL3*_KD *M. smegmatis* strain at different time points. (**A**) OD_600_ of the *mmpL3*_KD *M. smegmatis* strain at different ATc concentrations (ng/mL). (**B**) The viability (log10 CFU/mL) of the *mmpL3*_KD *M. smegmatis* strain at different ATc concentrations (ng/mL). The experiments were conducted in biological and technical triplicates. The error bars represent the SDs of the means.

The impact of *mmpL3* transcriptional repression on the growth curve and viability (colony forming unit (CFU)/mL) of the *mmpL3*_KD strain was examined in liquid media supplemented with ATc at concentrations ranging from 0 to 50 ng/mL. A defect in the growth curve and viability of the *mmpL3*_KD strain was evident at ATc concentrations ≥0.5 ng/mL. Moreover, a significant reduction in the optical density of 600 nm (OD_600_) of the culture was observed at 24, 36, and 48 h of induction with 1, 5, 10, and 50 ng/mL ATc (*p* < 0.05) compared to conditions without ATc (Figure 3A). In addition, the CFU/mL of *mmpL3*_KD rapidly decreased upon exposure to 1, 5, 10, and 50 ng/mL ATc (*p* < 0.05) within 4 h (Figure 3B). These significant reductions in CFU/mL corresponded to 3.65 ± 0.78-log10 CFU/mL.

Furthermore, the OD_600_ of the *mmpL3*_KD culture gradually increased after 24 h of ATc induction and was not significantly different from that in the absence of ATc at 72 and 96 h (Figure 3A). Similarly, the viability (CFU/mL) slowly increased and showed nonsignificant differences at 48, 72, and 96 h of ATc induction (Figure 3B). Additionally, the growth curve and viability of the pLJR962 control strain were comparable to those of the *mmpL3*_KD strain under conditions without ATc induction or with 0.1 ng/mL ATc (Supplementary Figure S2A,B). Moreover, the biofilm formation of *mmpL3*_KD was comparable to the pLJR962 control (*p* > 0.05). For pellicle formation, no pellicle was observed at high ATc concentrations (≥1 ng/mL), which could be due to defects in mycobacterial growth (Supplementary Figures S3 and S4).

### 2.4. Resensitization of the mmpL3_KD M. smegmatis Strain to Antimicrobial Agents

To evaluate whether *mmpL3* repression affects the susceptibility of the *mmpL3*_KD strain to non-MmpL3-dependent antimicrobial agents, the minimal inhibitory concentrations (MICs) of various antimicrobial agents against the *mmpL3*_KD strain were initially assessed. The MICs were determined as follows: 0.25 µg/mL for amikacin, 0.5 µg/mL for clarithromycin, 4 µg/mL for isoniazid, >512 µg/mL for ceftriaxone, >8 µg/mL for rifampicin, and 2 µg/mL for rifabutin. In the presence of 0.1 ng/mL ATc, the MICs of *mmpL3*_KD were not affected. However, the MICs decreased by one- to two-fold dilutions in the presence of 0.5 ng/mL ATc, which could be due to a growth defect resulting from *mmpL3* repression.

Therefore, the impact on the viability (CFU/mL) of the *mmpL3*_KD strain was assessed in the presence of 0.1 ng/mL ATc, which suppressed *mmpL3* expression but did not affect the growth of mycobacterial cells, and one-half of the MICs of the antimicrobial agents, which were 0.125 µg/mL for amikacin, 0.25 µg/mL for clarithromycin, 2 µg/mL for isoniazid, 256 µg/mL and 512 µg/mL for ceftriaxone, 4 µg/mL and 8 µg/mL for rifampicin, and 1 µg/mL for rifabutin. For rifampicin and ceftriaxone, two concentrations were tested because the MIC values exceeded the highest concentration tested in the experiment. One-half of the MIC of each antimicrobial agent reduced the CFU/mL of *mmpL3*_KD by approximately 0.17-log10 for isoniazid, 0.36-log10 for 256 µg/mL of ceftriaxone, 1.04-log10 for 512 µg/mL of ceftriaxone, 0.52-log10 for 4 µg/mL of rifampicin, 0.57-log10 for 8 µg/mL of rifampicin, and 1.20-log10 for rifabutin, compared to the no-drug control in the absence of ATc. No significant reduction in CFU/mL was observed with amikacin or clarithromycin.

**Figure 4:**
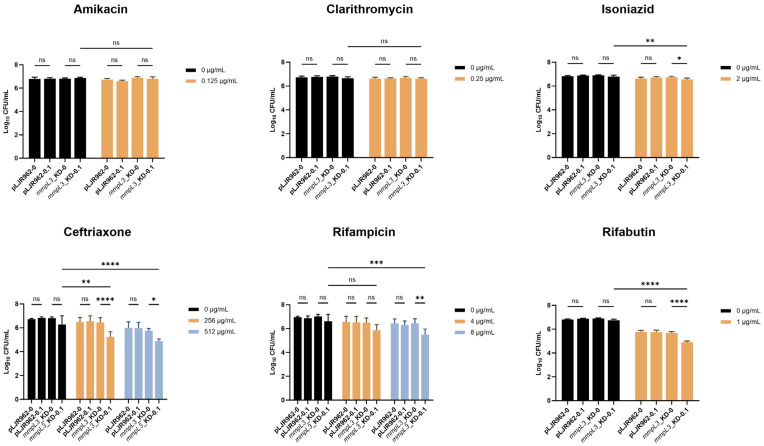
Repression of *mmpL3* with 0.1 ng/mL ATc resulted in resensitization to isoniazid, ceftriaxone, rifampicin, and rifabutin. The viability (log10 CFU/mL) of the *mmpL3*_KD and pLJR962 control *M. smegmatis* strains treated with antimicrobial agents in the presence or absence of 0.1 ng/mL ATc was determined in biological and technical triplicates. Statistical significance is indicated as * *p* < 0.05, ** *p* < 0.01, *** *p* < 0.001, and **** *p* < 0.0001, and “ns” indicates not statistically significant. The error bars represent the SDs of the means.

**Figure 5:**
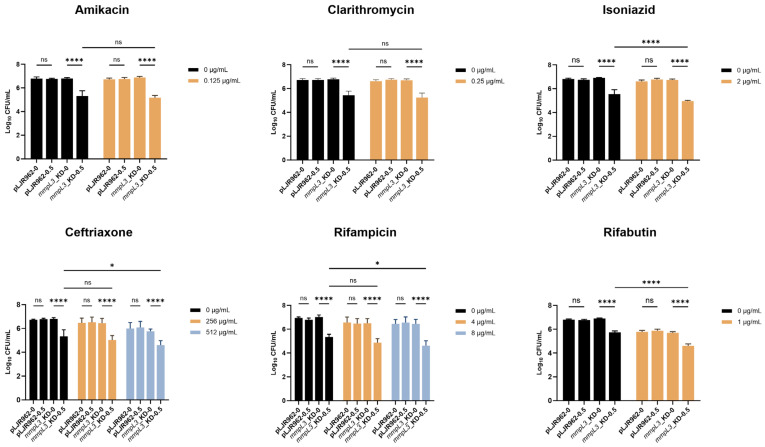
Repression of *mmpL3* with 0.5 ng/mL ATc significantly impacted *M. smegmatis* viability and antimicrobial susceptibility. The viability (log10 CFU/mL) of the *mmpL3*_KD and pLJR962 control *M. smegmatis* strains treated with antimicrobial agents in the presence or absence of 0.5 ng/mL ATc was determined in biological and technical triplicates. Statistical significance is indicated as * *p* < 0.05, **** *p* < 0.0001, and “ns” indicates not statistically significant. The error bars represent the SDs of the means.

Although the suppression of *mmpL3* with 0.1 ng/mL ATc did not alter the MIC, the combination of this ATc level and one-half of the MIC of certain antimicrobial agents resulted in a significant decrease in *mmpL3*_KD viability. Specifically, the viability of *mmpL3*_KD with 0.1 ng/mL ATc with isoniazid, ceftriaxone, rifampicin (8 µg/mL), and rifabutin was significantly reduced for 0.77 ± 0.35-log10 CFU/mL) compared with antimicrobial agents alone (*p* < 0.05) (Figure 4). However, clarithromycin and amikacin exhibited reductions of only 0.07 and 0.08-log10 CFU/mL, respectively (Figure 4). To assess whether greater *mmpL3* suppression can more effectively reduce mycobacterial viability, 0.5 ng/mL ATc was added to one-half of the MIC plates. As expected, the viability of the *mmpL3*_KD strain significantly decreased in the presence of 0.5 ng/mL ATc (*p* < 0.0001), showing a decrease of 1.47 ± 0.23-log10 CFU/mL compared to that in the absence of ATc, regardless of the antimicrobial agent concentration (0 µg/mL, one-half MIC) (Figure 5). Conversely, the addition of one-half MIC of isoniazid, rifabutin, ceftriaxone (512 µg/mL), or rifampicin (8 µg/mL) to *mmpL3*_KD with 0.1 or 0.5 ng/mL ATc significantly enhanced the decrease in bacterial viability (*p* < 0.05) compared to the condition with ATc alone (Figure 4 and Figure 5).

Additionally, induction of dCas9_Sth1_ without a sgRNA in the pLJR962 control did not increase the defects of growth or viability of *M. smegmatis* compared to treatment with antimicrobial agents alone (*p* > 0.05) (Figure 4 and Figure 5).

## 3. Discussion

MmpL3 functions as a transporter of trehalose monomycolate, a precursor of trehalose dimycolate [1,2]. *mmpL3* in *M. smegmatis* consists of 3042 base pairs encoding 1013 amino acids, serving as an ortholog of *rv0206c* in *M. tuberculosis*. Extensive studies have underscored the importance of *mmpL3* as an essential gene in *M. tuberculosis* [15,22], *M. smegmatis* [23], and *M. abscessus* [24]. Therefore, *mmpL3* has been identified as a promising target for developing new therapeutic agents against *M. tuberculosis* and NTM [3,4,5,6,25,26]. Previously, the synergistic effects of MmpL3 inhibitors [4,5,13,14] or *mmpL3* knockdown using various genetic tools [15,16,17] and certain antimicrobial agents have been investigated. However, these studies have demonstrated varying susceptibilities to non-MmpL3-dependent antimicrobial agents, which may be explained by differences in the level of *mmpL3* inhibition or repression achieved under different experimental conditions and in different organisms which can differ substantially in many aspects, limiting the direct application of findings from one to another. 

The CRISPRi system is easily constructed and requires only a single transformation, enabling the rapid generation of transcriptional knockdown strains of mycobacteria. In mycobacteria, CRISPRi has demonstrated effectiveness in suppressing drug resistance genes, particularly those associated with β-lactams, by targeting essential peptidoglycan synthesis genes such as *pbpB* and *cwlM* [20]. Furthermore, it has been utilized to target the rifampicin resistance gene ADP-ribosyltransferase (*arr*) [19]. This versatility makes CRISPRi a valuable tool for investigating resistance mechanisms and devising strategies to combat drug-resistant mycobacteria. In our study, the CRISPR-dCas9_Sth1_ system was used to repress *mmpL3* expression, and its impacts on *M. smegmatis* viability and susceptibility to non-MmpL3-dependent antimicrobial agents were evaluated. The induction of dCas9_Sth1_ with a high concentration of ATc did not adversely affect the growth or viability of *M. smegmatis* wild-type or vector control strains, as similarly demonstrated in a previous study [18]. Similar to a previous study [17], the depletion of MmpL3, which is below the levels required for the in vitro growth of mycobacteria, was rapidly achieved with low concentrations of ATc (0.5 ng/mL) in an ATc dose-dependent manner. In our study, this is attributed to the permissive PAM sequences utilized (NNAGAAA), which achieved a gene repression of up to 158.1-fold [18], and the sgRNA targeting the coding region of the *mmpL3* gene. Additionally, the gradual increase in growth was observed after 24 h of ATc induction. This phenomenon could be attributed to the degradation of ATc or the growth of the *mmpL3*_KD *M. smegmatis* escape mutants. Consequently, their potential influence on the results and subsequent statistical analysis cannot be disregarded. The emergence of strains unresponsive to the ATc inducer is consistent with findings from a previous study [17] and with other tetracycline-regulated promoters utilized in mycobacteria [27].

Importantly, this study investigated the effects of *mmpL3* repression on susceptibility to antimicrobial agents. Although a reduction in MIC and CFU/mL was not observed with an approximately 4% to 10% decrease in *mmpL3* expression in response to 0.1 ng/mL ATc, a significant reduction (*p* < 0.05) in CFU/mL was noted when this decreased expression was combined with half of the MICs of several classes of antibiotics, including rifamycin, β-lactam, and isoniazid, demonstrating a similar pattern. However, this reduction in viability did not meet the criteria for synergy, defined as a ≥2 log10 reduction in CFU compared to the antimicrobial agent alone [20]. Several hypotheses could explain these findings. For instance, the ATc concentration might be too low, resulting in insufficient *mmpL3* repression. Additionally, the emergence of nonresponsive escape mutants resistant to ATc could have contributed to these results. The significant decrease in CFU/mL observed in the rifamycin group, regardless of rifampicin or rifabutin, can be attributed to disruption of the mycolic acid in the cell wall, resulting in alterations in hydrophobicity [16]. This explanation could also apply to isoniazid, where *mmpL3* knockdown may enhance the inhibition of mycolic acid synthesis. In contrast, repression of *mmpL3* in *M. tuberculosis* using the CRISPRi system did not change the MICs of rifampin, isoniazid, or linezolid [17]. Additionally, the synergistic effect of SQ109, an MmpL3 inhibitor, against *M. tuberculosis* has been demonstrated with isoniazid and rifampicin but not with ethambutol and pyrazinamide in vitro [13]. With respect to ceftriaxone, *mmpL3*_KD was observed to result in improved susceptibility due to enhanced penetration of β-lactams facilitated by alterations in outer membrane assembly. However, no study has explored the effect of clarithromycin on *mmpL3* knockdown. One study demonstrated that specific single nucleotide polymorphisms (SNPs) within the *mmpL3* gene of *M. smegmatis* resulted in growth defects and susceptibility to ampicillin, rifampicin, and erythromycin, but not to chloramphenicol or kanamycin. This susceptibility arose because the depletion of *mmpL3* disrupts cell wall formation in mycobacteria [16]. These findings suggest that the synergistic mechanism of *mmpL3* repression or inhibition with antimycobacterial agents relies on optimal conditions, such as the level of repression and classes or concentration of antimicrobial agents used. Therefore, further studies are needed to elucidate the true mechanism involved.

In conclusion, these findings emphasize the utility of the CRISPRi-dCas9_Sth1_ system for selectively targeting and suppressing genes associated with different classes of antimicrobial resistance. Moreover, this approach has the potential to enhance studies focused on understanding of gene functions and resistance to antimicrobial agents. Additionally, the repression of the *mmpL3* gene in mycobacteria represents a promising strategy for treating mycobacterial infections, particularly those caused by drug-resistant strains.

## 4. Materials and Methods

### 4.1. Bacterial Growth and Culture Conditions

*M. smegmatis* MC^2^155 and its derivative strains were grown either in Middlebrook 7H9 (7H9) broth or Middlebrook 7H10 (7H10) solid media (BD Difco, Sparks, MD, USA) supplemented with 0.2% *v*/*v* glycerol (HiMedia, Maharashtra, India) and 10% ADC (5% BSA, 2% dextrose, 0.003% catalase) at 37 °C. The broth was further supplemented with 0.05% Tween 80 (Ajax Finechem, NSW, Australia) and incubated with shaking at 200 rpm. For cloning experiments, *Escherichia coli* DH5α was grown in Luria Bertani (LB) broth and agar at 37 °C. Kanamycin (GoldBio, St. Loius, MO, USA) was added as needed at concentrations of 50 µg/mL for *E. coli* and 25 µg/mL for *M. smegmatis*. Anhydrotetracycline (ATc, Sigma-Aldrich, St. Louis, MO, USA) was added at different concentrations as required for specific experiments.

### 4.2. Construction of sgRNA Expression Plasmids and mmpL3_KD Strains

*mmpL3* (*msmeg_0250*)-knockdown *M. smegmatis* (*mmpL3*_KD) was constructed using the CRISPRi system with the pLJR962 plasmid (Addgene no. 115162). This plasmid expresses both the targeting sgRNA and Sth1 dCas9 from a Tet repressor (TetR)-regulated promoter induced by ATc and contains a kanamycin selection marker. This plasmid integrates into the L5 *attB* site of mycobacterial chromosome when transformed into mycobacteria [18,21]. Briefly, the dCas9_Sth1_ protospacer adjacent motif (PAM) sequence against the reference *M. smegmatis* MC^2^155 gene was identified using a design tool (https://pebble.rockefeller.edu/tools/sgrna-design/, accessed on 1 November 2021). Then, 23 nucleotide sgRNA targeting sequences upstream of the PAM, 5′ NNAGAAA 3′, were extracted. The 5′ GGGA 3′ sequence was appended to the 5′ sequence of the sgRNA targeting sequence for cloning the sgRNA [21]. The sgRNA oligos targeting the *mmpL3* gene were *mmpL3*_T: 5′ GGGAGCGACAGACTGGCTGCCCTCGTC 3′ and *mmpL3*_B: 5′AAACGACGAGGGCAGCCAGTCTGTCGC 3′, which were previously designed to target the nontemplate strand of *mmpL3* [21]. The sequence of sgRNA is specific to *mmpL3* of *M. smegmatis* MC^2^155 without complementarity to other sites in the genome.

These sgRNA oligos were annealed, ligated into a BsmBI-digested CRISPRi backbone (pLJR962), and transformed into *E. coli* DH5α, which was selected on LB agar supplemented with kanamycin. Purified sgRNA-pLJR962 plasmids from *E. coli* were verified using Sanger sequencing with the pLJR962-965-SS primer: 5′ TTCCTGTGAAGAGCCATTGATAATG 3′. Next, sequence-verified plasmids (100 ng) and pLJR962 plasmids without sgRNA (empty vector control) were electroporated at 25 kV and 25 µF with 1000 W resistance into electrocompetent *M. smegmatis* MC^2^155 strains, which were prepared as previously described [21]. The transformants were selected on 7H10 agar supplemented with 25 µg/mL kanamycin in the presence or absence of 50 ng/mL ATc to determine the bacterial viability of the *mmpL3*_KD strains. Subsequently, the presence of the CRISPRi-dCas9_Sth1_ construct in *M. smegmatis* was confirmed by PCR.

### 4.3. Determination of the Essentiality of the mmpL3 Gene in M. smegmatis

Mid-log phase cultures of both *mmpL3*_KD and pLJR962 control *M. smegmatis* strains grown in 7H9 broth supplemented with 0.2% glycerol, 0.05% Tween 80, 10% ADC, and 25 µg/mL kanamycin were diluted to an OD_600_ of 0.1. Subsequently, these cultures were serially diluted 10-fold (10^−1^–10^−5^). Five microliters of the diluted cultures were then spotted on 7H10 agar plates containing different concentrations of ATc (0, 0.1, 0.5, 1, 5, 10, and 50 ng/mL) and incubated at 37 °C for 3 days.

### 4.4. Bacterial Growth Curve and Viability Count

The mid-log phase cultures of the *mmpL3*_KD and pLJR962 control *M. smegmatis* strains were prepared at an OD_600_ of 0.1 and induced with various concentrations of ATc: 0, 0.1, 0.5, 1, 5, 10, and 50 ng/mL. The cultures were then incubated, and the OD_600_ was measured at different time points from 0 to 96 h. For viability assessment, the CFU/mL of cultures were determined by collecting cultures both with and without ATc at various time points ranging from 0 to 96 h. Each culture condition was serially diluted (10^−2^–10^−8^), and 20 µL of the diluted samples was plated and incubated at 37 °C for 3 days or until visible colonies were observed to determine the CFU/mL. All experiments were conducted in biological and technical triplicates.

### 4.5. Determination of the Minimal Inhibitory Concentration and Mycobacterial Viability

The *M. smegmatis mmpL3*_KD and pLJR962 control strains were cultured on 7H10 agar plates and incubated at 37 °C until visible colonies were observed (2–3 days). Drug susceptibility tests were conducted using a broth microdilution method following the Clinical & Laboratory Standards Institute (CLSI) recommendations to determine MIC values [28]. Briefly, 100 µL of bacterial suspensions (approximately 5 × 10^5^ CFU) were added to each well of sterile flat bottom 96-well plates (Jet Biofil, Guangzhou, China) that contained drugs at 2-fold serial dilutions. The antimicrobial agents tested included ceftriaxone (1–512 µg/mL), rifampicin (0–8 µg/mL), amikacin (1–6 µg/mL), rifabutin (0.25–8 µg/mL), isoniazid (0.25–8 µg/mL), and clarithromycin (0.06–16 µg/mL) with the presence or absence of ATc. Then, the plates were incubated at 30 °C for 2–3 days until a sufficient positive control (no antimicrobial agent) was obtained. *Mycobacterium peregrinum* ATCC700686 served as the quality control strain.

The impact of the ATc inducer and antimicrobial agents on the viability of the *mmpL3*_KD and pLJR962 control *M. smegmatis* strains was evaluated. Five microliters of 10-fold serial dilutions (10^−1^–10^−6^) of mid-log phase cultures were plated on 7H10 agar containing one-half of the MIC of the respective antimicrobial agents in the presence or absence of 0.1 and 0.5 ng/mL ATc. The plates were then incubated at 37 °C until visible colonies were observed to determine the CFU/mL. All experiments were conducted with biological and technical replicates.

### 4.6. RNA Extraction, cDNA Synthesis, and RT-qPCR

To investigate the strength of the transcriptional repression, mid-log phase cultures of the *mmpL3*_KD and pLJR962 control *M. smegmatis* strains were initiated at an OD_600_ of 0.1 in 7H9 broth supplemented with ADC, 0.05% Tween 80, and 25 µg/mL kanamycin. These cultures were then induced with varying concentrations of ATc (0, 0.1, 0.5, and 1 ng/mL) for 12 h. RNA extraction was conducted as previously described [29]. Briefly, bacterial cells harvested at 0 and 12 h after ATc induction were collected via centrifugation at 2000× *g* at 4 °C for 5 min and then resuspended in 1 mL of TRIzol solution (Invitrogen, Waltham, MA, USA) on ice. Then, the cells were disrupted using acid-washed glass beads (150–212 µm) (Sigma-Aldrich, St. Louis, MO, USA) for three cycles of 30 s at a speed of 6.5 m/s using a FastPrep-24 instrument (MP Biomedicals, Santa Ana, CA, USA).

RNA was purified using 300 µL of chloroform: isoamyl alcohol (24:1) followed by precipitation using 270 µL of isopropanol and 270 µL of 1.2 M NaCl at 4 °C for 3–4 h. The RNA pellet was then washed twice with 75% ethanol. Furthermore, the RNA samples were treated with a Turbo-DNA-free Kit (Invitrogen, Waltham, MA, USA) according to the manufacturer’s instructions. cDNA was synthesized using iScript™ Reverse Transcription Supermix (Bio-Rad Laboratories, Hercules, CA, USA) following the manufacturer’s instructions. qPCR was conducted to quantify the expression of *mmpL3* and *sigA* using Luna^®^ Universal qPCR Master Mix (New England Biolabs, Ipswich, MA, USA) with 50 ng of cDNA and 0.12 µL of *sigA* primers (*msmeg*_*sigA*_F: 5′ GACGACGACATCGACGAG 3′ and *msmeg*_*sigA*_R: 5′ GTCAGCTCGGCGTCTTTG 3′) and *mmpL3* primers (*msmeg*_*0250*_F: 5′ TCGATCAGGTGGTCAAGGA 3′ and *msmeg*_*0250*_R: 5′ GCAGATCCTGCGTCTTCAT 3′), respectively, in a final volume of 20 µL per reaction.

The qPCRs were run on a QuantStudio 5 Real-Time PCR system (Applied Biosystems, Waltham, MA, USA) with the following cycling conditions: an initial denaturation step at 95 °C for 60 s, followed by 45 cycles of denaturation at 95 °C for 15 s and annealing and extension at 60 °C for 30 s. To normalize the expression levels of *mmpL3*_KD, the mRNA levels of the reference *sigA* gene were used. The relative gene expression levels were analyzed relative to the expression of the pLJR962 control. Additionally, melting curve analysis was performed to verify the specificity of the qPCR amplification. All experiments were conducted in biological and technical triplicates.

### 4.7. Statistical Analysis

The statistical significance of differences in viability and gene expression at different ATc concentrations or time points was assessed using one-way or two-way ANOVA, as appropriate. A *p* value less than 0.05 was considered to indicate statistical significance. Statistical analysis and graph were generated using GraphPad Prism version 10.0.0 for Windows.

## Data Availability

No datasets were generated during the study. All the data analyzed in this study are included in this published article.

## Supplementary Materials

The following supporting information can be downloaded at: https://www.mdpi.com/article/10.3390/antibiotics13060483/s1, Supplementary Materials and Methods: Biofilm and pellicle formation; Figure S1: Essentiality of the *mmpL3* gene in the *mmpL3*_KD and pLJR962 control *M. smegmatis* strains; Figure S2: Growth curve and viability of the *mmpL3*_KD and pLJR962 control *M. smegmatis* strains at different time points; Figure S3: Biofilm formation of the *mmpL3_*KD and pLJR962 control *M. smegmatis* strains assessed by a quantitative crystal violet assay; Figure S4: Pellicle formation of the *mmpL3*_KD and pLJR962 control *M. smegmatis* strains observed on day 3 of incubation. Reference [30] is cited in the Supplementary Materials.
