# Supplementary material for: CRISPR Interference-Mediated Silencing of the mmpL3 Gene in Mycobacterium smegmatis and Its Impact on Antimicrobial Susceptibility
Source: Antibiotics (Basel). 2024 May 24;13(6):483. doi: 10.3390/antibiotics13060483 (PMC11200583; doi:10.3390/antibiotics13060483)
Supplement: Supplementary file 1 [file antibiotics-13-00483-s001.zip › antibiotics-3010202-supplementary.pdf]

Supplementary Material

CRISPR interference-mediated silencing of the *mmpL3* gene in *Mycobacterium smegmatis* and its impact on antibiotic susceptibility

Yonita Yuliani[1], Azizah Fitriana Nurul Ilmi[1], Suthidee Petsong[2], Ajcharaporn Sawatpanich[2], Sunisa Chirakul[2,3], Tanittha Chatsuwan[2,3], Tanapat Palaga[4], Suwatchareeporn Rotcheewaphan[2,3]*

[1] Medical Sciences, Faculty of Medicine, Chulalongkorn University, Bangkok, Thailand
[2] Department of Microbiology, Faculty of Medicine, Chulalongkorn University, Bangkok, Thailand
[3] Center of Excellence in Antimicrobial Stewardship, Chulalongkorn University, Bangkok, Thailand
[4] Department of Microbiology, Faculty of Science, Chulalongkorn University, Bangkok, Thailand

Supplementary Data

Materials and methods

Biofilm and pellicle formation

M. smegmatis MC^{2}155 derivative strains were cultured on Middlebrook 7H10 solid medium (BD Difco, Sparks, MD, USA) supplemented with 0.2% v/v glycerol (HiMedia, Maharashtra, India), 10% ADC, and 25 µg/mL kanamycin (GoldBio, St. Loius, MO, USA) at 37°C until visible colonies were observed. A single colony from each strain was then inoculated into Middlebrook 7H9 broth (BD Difco, Sparks, MD, USA) supplemented with 10% ADC and 0.05% Tween 80 (Ajax Finechem, NSW, Australia), along with 25 µg/mL kanamycin, and incubated for 24 hours with shaking at 200 rpm and 37°C. Biofilm and pellicle formation were assessed as previously described [30] with slight modifications. Log-phase cultures of *M. smegmatis* were adjusted to an OD_{600} of approximately 0.1 in Sauton medium (HiMedia, Maharashtra, India) containing varying concentrations of ATc (0 to 50 ng/mL) and 25 µg/mL kanamycin. For pellicle formation assays, 2 mL aliquots of the diluted culture were incubated in glass tubes without shaking at 37°C for 3 days. For biofilm formation assays, 200 µl aliquots of the diluted cultures were inoculated into 96-well polystyrene plates (Jet Bio-filtration, Guangzhou, China) in replicates of six and incubated at 37°C for 5 days.

After incubation, the 96-well plates were washed twice with water, stained with 0.1% crystal violet for 30 minutes, and then washed twice with water. Bound crystal violet was solubilized with 30% acetic acid, and the absorbance was measured at 595 nm.

Statistical analysis

The statistical significance of biofilm formation under different ATc concentrations was assessed using two-way ANOVA. A p value less than 0.05 was considered to indicate statistical significance. Statistical analysis and graph were generated using GraphPad Prism version 10.0.0 for Windows.

Supplementary Figures

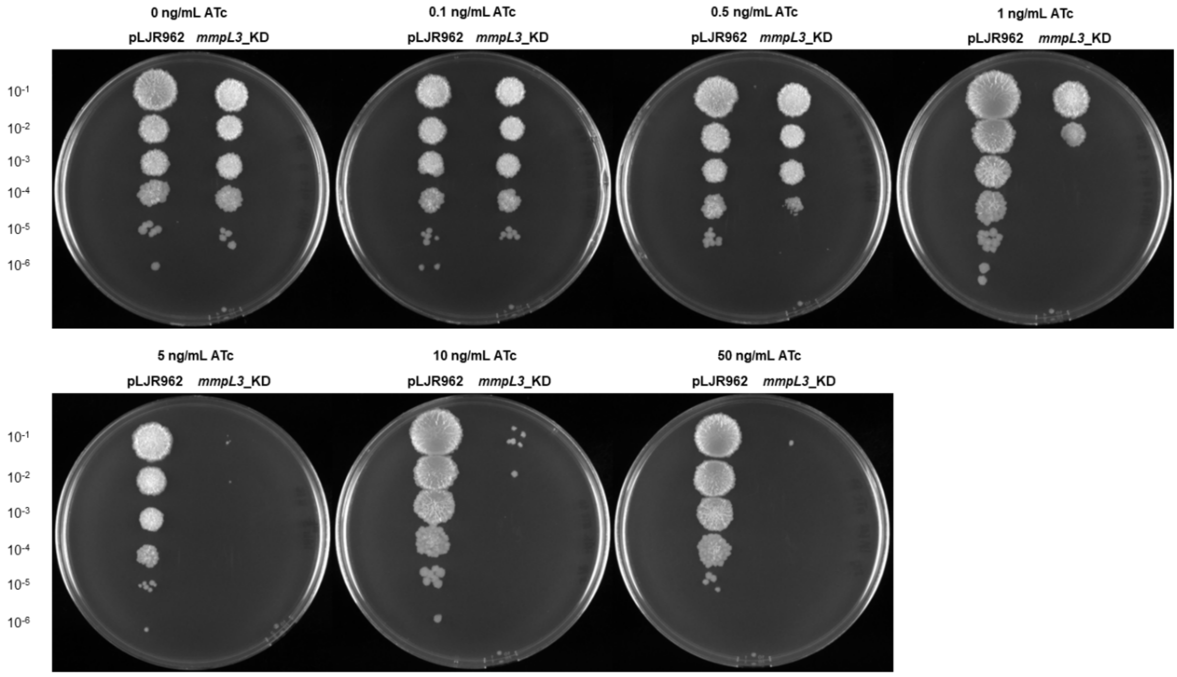

Figure S1. Essentiality of the *mmpL3* gene in the *mmpL3*_KD and pLJR962 control *M. smegmatis* strains. Serial dilutions (10^{-1} to 10^{-6}) of log-phase cultures of the *mmpL3*_KD and pLJR962 control *M. smegmatis* strains were plated on 7H10 agar in both the absence and presence of varying concentrations of ATc (0, 0.1, 0.5, 1, 5, 10, or 50 ng/mL).

A

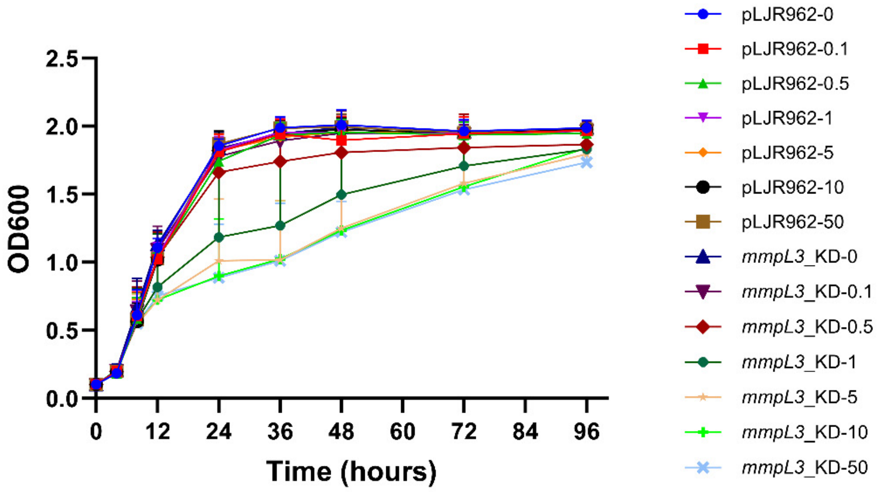

B

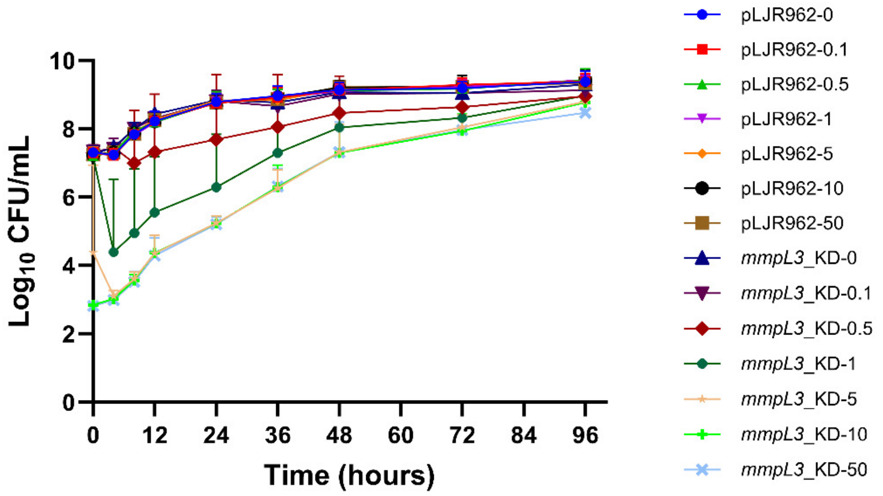

Figure S2. Growth curve and viability of the *mmpL3*_KD and pLJR962 control *M. smegmatis* strains at different time points. (A) The optical density at 600 nm (OD_{600}) of the *mmpL3*_KD and pLJR962 control *M. smegmatis* strains at different ATc concentrations (0, 0.1, 0.5, 1, 5, 10, or 50 ng/mL). (B) The viability (log10 CFU/mL) of the *mmpL3*_KD and pLJR962 control *M. smegmatis* strains at different ATc concentrations (0, 0.1, 0.5, 1, 5, 10, or 50 ng/mL). The experiments were conducted in biological and technical triplicates. The error bars represent the SDs of the means.

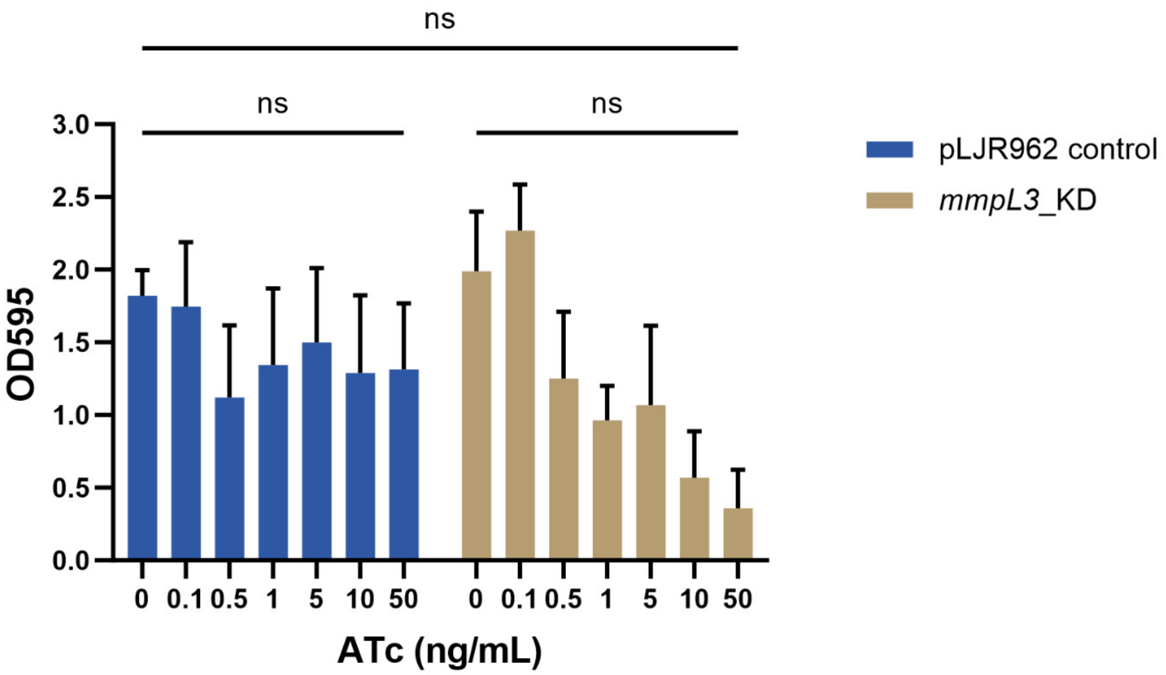

Figure S3. Biofilm formation of the *mmpL3*_KD and pLJR962 control *M. smegmatis* strains assessed by a quantitative crystal violet assay. A decrease in the biofilm mass of the *mmpL3*_KD *M. smegmatis* strain was observed after induction with 0.5 ng/mL to 50 ng/mL ATc ($p > 0.05$). The experiments were conducted with six replicates. "ns" indicates not statistically significant. The error bars represent the SDs of the means.

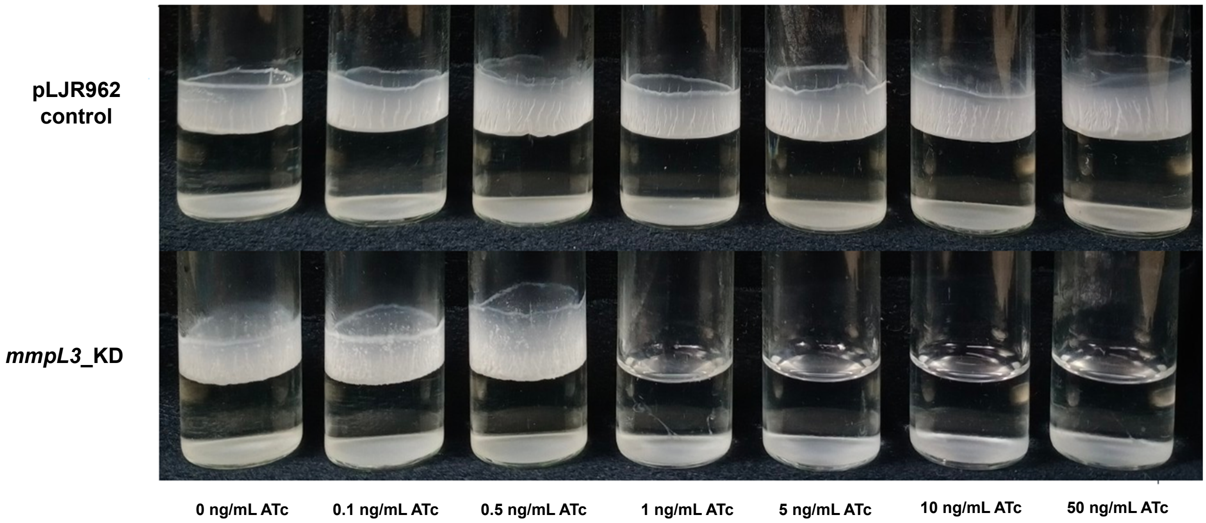

Figure S4. Pellicle formation of the *mmpL3*_KD and pLJR962 control *M. smegmatis* strains observed on day 3 of incubation. Pellicle formation of the *mmpL3*_KD *M. smegmatis* strain decreased in the presence of 1 ng/mL to 50 ng/mL ATc compared to the pLJR962 control strain.
